# Online Peer-Support Group’s Role in Addressing Filipino Domestic Workers’ Social Support Needs: Content and Social Media Metrics Analysis

**DOI:** 10.3390/ijerph19159665

**Published:** 2022-08-05

**Authors:** Qijin Cheng, Christine Vicera

**Affiliations:** Department of Social Work, The Chinese University of Hong Kong, Hong Kong, China

**Keywords:** migrant domestic worker, wellbeing, mental health, social support, peer support, online, social media, Facebook

## Abstract

The study aimed to examine what types of social support a prominent online peer support group has provided to Filipino domestic workers (FDWs) in Hong Kong (HK), how impactful each type of support was, and to what extent the support could address group members’ expressed needs. Posts published from 1 September 2021 to 31 December 2021 in one of the largest online peer-support groups for FDWs in HK and their meta-data were extracted from Facebook. Thematic content analysis was performed to extract relevant information from the posts. Descriptive statistical analysis on the social media metrics was conducted to measure post impacts. A total of 599 posts published over the study period can be categorized to provide informational (76.67%), emotional (33.56%), and instrumental support (13.52%). Posts including emotional support were often more impactful. A total of 58 posts responded to requests explicitly expressed by individual FDWs, of which 13 required external resources. The online peer-support group acts as a new channel to raise FDWs’ awareness of important issues related to their well-being, to encourage and facilitate them to seek formal and informal help, and to keep them emotionally attended to. Formal support services should recognize and collaborate with them and support their long-term sustainability.

## 1. Introduction

Migrant domestic workers (MDWs) have been instrumental in providing care for local families and enabling more of the local population to join the workforce worldwide. Hong Kong (HK) is no exception [1]. According to the HK Immigration Department, in 2021, there were 339,451 MDWs working in the city, 191,783 (56.5%) of whom were from the Philippines [2]. Since the HK population is rapidly aging, the local government predicts the demand for MDWs in HK to reach 600,000 by 2047 [3]. MDWs in HK, similar to in other places, are found to have a high prevalence of physical and mental health issues and are often marginalized from formal healthcare and social services [4,5,6]. Their vulnerability has been further exacerbated by COVID-19, with many of them being demanded an heavier workload, restricted to go out, or even forced to sleep on the street after testing positive [7,8,9].

To address MDWs’ mental health needs, a full spectrum of social support, including informational, instrumental, and emotional support, should be provided [10,11,12]. Theoretically, social support, coming from both formal and informal sources, can provide a positive effect, a sense of predictability and stability in one’s life situation, a recognition of self-worth, a buffer from the potentially pathogenic influence of stressful events, and help to enhance one’s resilience [13,14,15]. Empirically, research suggests that MDWs seldom make use of formal resources, such as healthcare and social services, but often turn to informal support when encountering any challenges [10]. This phenomenon is consistent with research on other migrant populations as informal social networks, especially peer support networks, are found to be their primary source of support [16,17]. Therefore, informal social support has been recommended by the World Health Organization (WHO) as an important approach to promote migrants’ mental health [18]. 

In the digital era, online social media has become a vital platform for people to stay connected and share information; this is no exception for MDWs. The widespread availability of free WiFi hotspots throughout public areas in HK [19], the demand from employers to stay in contact, and the affordable price of smartphones have contributed to the widespread use of smartphones by MDWs in HK [20,21,22]. Subsequently, an increasing number of social media-based peer support groups have become available for MDWs in HK. In May 2017, Rodelia Pedro Villar, a Filipino domestic worker (FDW), created a Facebook (FB) account under the alias “Lovely Corner” and a public page—Domestic Workers Corner HONG KONG (DWC HK). Because of the founder’s nationality, the posts in this page are written in either English or Tagalog. Therefore, its followers are mainly FDWs in HK. This page quickly became one of the largest online peer-support groups for FDWs in HK. As of September 2021, when the present study started data collection, this public page had over 120,000 followers, equivalent to almost 70% of the FDWs working in HK in 2021. In 2020, Rodelia received the Spirit of Hong Kong Award in recognition of the contributions her and the 20 volunteer administrators (also known as admins) behind DWC HK have made for the MDW communities in Hong Kong [23]. 

In spite of these developments, research on peer support for MDWs in HK has not caught up. Previous studies on peer support for MDWs often only consider offline settings (and sometimes acquaintances affiliated with religious groups), but seldom consider peers met in online anonymous settings [24]. To the best of our knowledge, no study has specifically examined how the emerging online peer networks play a role in providing social support to MDWs. In addition, previous research has noted both advantages and shortcomings of peer support through conventional social networks. For example, the advantages include high accessibility and responsiveness to peers’ needs, enhancing individual and community’s problem-solving competence, as well as promoting a sense of community [11,25]. On the other hand, the limitations or shortcomings include worries about gossip and rumors spread by peers [10,26,27], limited support for informational and instrumental support [10] or even being exposed to misinformation or mal-coping strategies [11,12,28], negative emotion contagion [27,29], power struggles and conflicts in the groups [16], and lack of support from culturally competent mental health professionals [30]. It is not clear to what extent these benefits and limitations still hold true to online peer-support groups, and whether new benefits and drawbacks emerged in the new context.

DWC HK offers an opportunity for us to map out the extent to which online peer support groups can provide social support to FDWs. In addition, due to the clear documentation of the online discussions, it might be the first time that voices of such a large group of FDWs can be heard and studied by researchers in a natural environment. This can complement the methodological limitations of previous research on MDWs which heavily relied on structured questionnaire surveys or qualitative interviews. Structured questionnaire surveys often use standardized scales to measure the prevalence of common mental or physical health issues. However, standardized scales may not be able to capture the nuance in their needs. Qualitative interviews are supposed to address the limitation of structured surveys by discovering in-depth experiences. However, published qualitative studies on MDWs’ mental health and peer support are often limited by a small sample [24].

Therefore, the present study focused on DWC HK, a FB-based group, to examine what role it has played in providing social support to its group members. Specifically, our analysis aimed to map out what specific kinds of social support were proactively or reactively provided, which types of support was more impactful on the group members, which specific needs as expressed by the group members were well addressed by the group, and which specific needs required further external resources to be attended to. 

## 2. Materials and Methods

With the page founder’s consent, we have downloaded all posts published from 1st September–31 December 2021 on DWC HK and their meta-data, including number of comments, shares, and reactions (reactions can be further broken down to Like, Love, Wow, Haha, Support, Sorry, and Anger) through “Export Comments,” an online service which has been used by previous studies to extract FB data [31,32]. All posts were published in public mode and no personal identifiers were collected by this study. The study has obtained ethical approval from our university’s IRB.

After downloading, thematic content analysis [33,34,35] was conducted by both authors to extract themes related to the research questions. Both authors have read and re-read the data multiple times to gain familiarity and consulted the founder of DWC HK from time to time when having any uncertainty with understanding the content. The thematic content analysis started with semi-structured coding by applying the conceptual framework of classifying social support into three basic dimensions: informational, instrumental, and emotional support [13,16,36]. The operational definitions and example posts can be read in Table 1. Meanwhile, the researchers allowed new categories and sub-categories to emerge to discover more nuanced findings. 

An iterative reading and re-reading procedure was implemented to develop the content coding scheme. At first, 50 posts were randomly selected from the database for the two researchers (QC and CV) to separately read and conduct the semi-structured coding. Because most of the posts were written in Tagalog or a mixture of Tagalog and English, CV, who is bilingual, translated those posts into English for QC to read. The coding results were compared and discussed, whereby a refined coding scheme was developed. When the two could not resolve a disagreement, discussions with admins of DWC HK were conducted to gain their insights. Then, the two applied the refined coding scheme to separately code another 50 posts that were randomly selected. The coding results were compared again to check the inter-rater consistency and the disagreements were discussed to find consensus. After three rounds of such exercise, the two researchers’ inter-rater reliability, examined by Cohen’s Kappa [37,38], improved from 0.76 to 0.89 to full agreement. Then, the coding scheme was confirmed. It was found that the three categories of social support needs can well summarize the social support categories identified in the posts, while our open coding identified more specific issues or circumstances that triggered those social support categories. Then, CV applied the finalized coding scheme to code all the posts. A total of 50 final coding results were randomly selected and double checked by QC to confirm full agreement. The coding results were communicated to and confirmed by the admins of DWC HK for member checking [39].

Although all posts were published by the FB page admins, some posts started with a sentence explicitly indicating that the following content was posted per request from an anonymous member. Normally, members of the FB page would send private messages to the page admins to ask questions. When the admins could not answer their questions, they publish the questions on the page with the senders’ consent. Therefore, those posts are labelled as “reactive posts”, which differ from regular “proactive posts” created by the admins themselves. Regarding “reactive posts”, we further read into the comments to confirm whether the needs were well addressed. If the person who expressed the need or the group admin left a comment indicating that the need has been attended to, this need was coded as “addressed”, otherwise, “not addressed”.

To check the impact of a specific support as expressed in a post, the numbers of comments, shares, and reactions to each post, were examined, which are common indicators of FB posts’ engagement effects and impacts [40,41,42]. Descriptive statistical analysis was conducted to report the results. Since most data were not normally distributed, median, instead of mean, was reported to indicate the data centrality.

## 3. Results

A total of 599 posts were published in DWC HK during the four-month period, which received 57,260 comments, 37,624 shares, and 268,532 reactions. The median number of posts per day was 6 (range: 1–19), and the median number of comments, shares, and reactions received per post was 19 (range: 0–2100), 21 (range: 0–1171), and 104.5 (range: 0–3842), respectively. 

### 3.1. Types of Support Provided

A total of 516 posts (86.14%) only attended to one type of social support. Among them, the majority provided purely informational support (n = 324), followed by emotional support (n = 143) and instrumental support (n = 49) such as organizing a winter clothes drive, filling up complicated legal documents, distributing free COVID-19 testing kits, organizing skills training programs, and providing a hotline to answer peers’ emergency calls. The other 83 posts (13.86%) attended to more than one need, a common combination being informational and emotional support (n = 49). Details and examples can be found in Table 1. When counting each type of social support individually in order of descending frequency, informational support (n = 400, 76.67% of total) scored the highest, followed by emotional support (n = 201, 33.56%), and instrumental support (n = 81, 13.52%).

Of the total posts, 509 (84.97%) were “proactive posts” created by the page admins, the remaining 90 (15.03%) being “reactive posts” posted by admins on behalf of anonymous FDW members. As shown in Table 2, our analysis further identified specific issues or circumstances that triggered the posts in the peer-support group. These diverse issues and circumstances cover almost every aspect of a FDW’s life in HK. Upon initial comparison, these issues and circumstances fall under the scope of service provided by local formal support resources catered to MDWs. Issues include queries pertaining to physical health, the processing of legal documents, skills training, prevention of scams and crimes, political participation, financial literacy, mental health, and labor rights. As shown in Examples 10 and 13 in Table 1, the group admins have established collaboration with public services (e.g., Consulate General of the Philippines in HK, Equal Opportunity Commission) and social services (e.g., Mission for Migrant Workers, HELP for Domestic Workers, PathFinders, Enrich, Uplifters, and other NGOs that support MDWs). The group admins have either reminded the members to make good use of those services (Examples 1, 2, and 10), offered more hands-on support to facilitate access to those resources (Example 11 in Table 1), or even collaborated with some NGOs to organize webinars or training workshops (Example 13 in Table 1).

Meanwhile, many concerns were unique to the group and fell out of the scope offered by formal support services. For example, the top three categories that most of the posts fall under include miscellaneous daily life issues, greetings, prayers, and sharing of funny or heartwarming things (e.g., Examples 3 and 4 in Table 1), and activities regularly organized by group members (e.g., Example 6 in Table 1). Although the posts in most categories were dominantly proactive, there are two exceptions—namely, lost and found (100% responsive posts, e.g., Examples 5 and 8 in Table 1) and matters related to employers (54.55% responsive posts), mainly about helping employers find replacements when a FDW is about to terminate her contract. Those two categories, as well as the category about community members’ deaths, were also not normally covered by formal support resources. Combining these categories together, 336 posts (56.09% of the total) covered needs that were not normally addressed by formal support. 

### 3.2. Category of Social Support Perceived as More Impactful

As shown in Table 1, the 143 posts offering emotional support received the most comments (median = 205.5) among all the categories, followed by posts combining informational and emotional support (median = 89) and the ones combining informational, instrumental, and emotional support (median = 78.5). The 49 posts that combined informational and emotional support received the most shares (median = 446) and reactions (median = 1613). In terms of shares, the posts only providing emotional support (median = 208) ranked second highest, and those providing informational support (median = 136) ranked third. In terms of reactions, the posts combining informational, instrumental, and emotional support (median = 1368) ranked the second, and those providing emotional support ranked third (median = 1133). 

When we read into the individual posts, the post (Example 4 in Table 1) that received the greatest number of comments (n = 2100) was one that showed appreciation with a picture taken by a group member. The post included not only a beautiful picture but also some emojis to express the admin’s emotional connection with the person who took the picture. Most of the comments were replies from other group members sharing photos they themselves have taken of Hong Kong’s different landscapes as well as questions about where the photos were taken. This post also received 40 shares and 1873 reactions, including 1450 Likes, 334 Loves, 4 Supports, 83 Wows, and 2 Hahas. 

The post (Example 1 in Table 1) that received the greatest number of shares (n = 1171) was a post about how to register for the Overseas Employment Certificate (OEC) on the website of the Philippines Overseas Employment Administration (POEA). For FDWs, this document is important, as without it, FDWs are not allowed to leave the Philippines legally. The post provided a detailed illustration of how to fill in the online registration form. As shown in its comments, group members found that the information effectively addressed their pain points when using the online registration platform.

The post (Example 12 in Table 1) which received the greatest number of reactions (n = 3842) was the page founder’s announcement on the changes regarding some of the FB groups she originally had founded. As informed by the founder, several of her peer admins had disagreements on the development and management of those groups, and thus, claimed control over some groups. Therefore, she declared that those groups are no longer associated with her and stressed that she will continue to offer support to the members through the remaining groups and her hotline. The post received 2550 Likes, 1050 Loves, 13 Sorrys, 226 Supports, and 3 Wows, but no Anger. 

### 3.3. Needs Addressed

A total of 58 posts included explicit requests for help, including questions asked by the admins or other group members through the admins, which suggests that the admins could not answer those questions or requests by themselves. A total of 45 of them were well addressed by the comments or replies made by the peer members, including 15 about daily life issues (e.g., where to exchange Philippine pesos to Hong Kong dollars, cell phone service providers in Hong Kong, boarding house information, where to buy household items), eight about physical health issues (e.g., surgical procedure for hemorrhoids, reminders to take rest to avoid getting physically ill), six about political participation (e.g., instructions on how to register for overseas voting), four related to employers (e.g., job openings, questions about finding new employers or waiting to finish contract first), four about lost belongings, two about religious issues (e.g., location of churches), one about legal documents, and one about love scams. 

As for the remaining 13 posts, we could not identify satisfying answers in the comments and replies. These posts included six posts which reported lost belongings or person, four related to employers (difficulty finding a new employer after termination of contract, being overworked by employers, finding a replacement to finish work contract), one about legal documents (what documents are needed for domestic workers to fly back to the Philippines amidst COVID-19 on short notice), one about financial literacy and mental health (members feeling depressed due to financial issues), and one about physical health (finding a gynecologist in Hong Kong) and daily life issues (finding insurance companies to cover the cost).

As shown in Table 3, it is noteworthy that the expressed needs that were addressed appeared to receive more comments than those not addressed, but those not addressed received more shares and reactions. 

## 4. Discussion

The study findings highlight that DWC HK has added a new channel to provide a full spectrum of social support. Consistent with previous findings about conventional peer support for MDWs, the online group is found to be timely and responsive, which can promote a better sense of community. DWC HK continuously published at least one post per day during the study period. Its average monthly engagement rate during the study period was 75.7%. This was calculated by dividing the sum total of comments, shares, and reactions by the number of page likes, which was significantly higher than 0.47%, the average monthly engagement rate of general FB public pages from September to December 2021 [43]. The results demonstrate the commitment and competence of the page admins to engage their community. The following part will discuss some specific findings by relating them to the research questions and comparing them with previous literature.

### 4.1. Informational, Instrumental and Emotional Support

Compared to previous studies on conventional peer support, the present study found that the online peer support group is not only active in providing emotional support but also informational and instrumental support. Although one recent study noted how misinformation and rumors about COVID-19 is spread through some MDW social networks [11], the posts in DWC HK appeared to correct misinformation and connect members with information from more credible sources such as government departments or NGOs. In addition, although another study noted how FDWs often contacted their consulate or other formal organizations for tangible support [5], our findings demonstrated an alternative path for them to acquire tangible support, which is directly through hands-on support provided by admins of DWC HK or being referred by DWC HK to those formal organizations. Either way, the online group can facilitate more FDWs to be connected with NGOs and other community resources, and can potentially enhance FDWs’ problem-solving competence. 

In addition, some of the instrumental support offered by the online group fills in the service gaps of formal support, such as technical support for FDWs to access online resources provided by formal organizations. It is worth highlighting that a few posts repeatedly reminded the group members that they can call a hotline offered by the page founder in the event of an emergency. As shown in the results, the founder was overwhelmed by the calls, so she had to ask the group members to contact NGOs when she could not answer their calls. The results suggest a great demand for this kind of peer support service, which, however, has never been provided with formal support or enhancement by any formal organization in HK. 

The findings also show that a big proportion of the posts addressed miscellaneous daily life issues, ranging from lost belongings, sharing experiences of being scammed, to asking for help when diagnosed with a serious disease. These suggest that, even though members are anonymous in the online group, they are comfortable enough to ask for and provide casual support to each other. In fact, previous studies found that some FDWs were reluctant to seek help from their peers because of worries about being judged or gossiped by their peers [10,26,27]. The anonymity of an online setting might have addressed this concern and created a more comfortable environment for FDWs to disclose their difficulties.

Our findings also note an increased number of comments, shares, and reactions when the online group incorporated elements of emotional support into the posts which mainly provided informational or instrumental support. This result suggests that, by first empathizing with and caring for their peers, the online group can enhance the impacts of their informational and instrumental support. In turn, we argue that the informational and instrumental support provided by the online group can be considered an indirect form of emotional support. That is, the sharing of information or volunteering services reflects the care they have for the group members and can, therefore, enhance the connectivity among the members. 

### 4.2. Proactive and Reactive Support

The results showed a bigger proportion of proactive support than reactive support. This can be partially attributed to the style of the group admins, who are often more resourceful and willing to share their resources to create an enabling environment. 

Previous studies noted that some conventional peer support groups promoted endurance or praying as coping strategies for hardship, which are found to be ineffective to address MDWs’ mental health needs [11,12,28]. As opposed to those conventional groups, DWC HK admins repeatedly encouraged the group members to seek help from formal services and sometimes collaborate with NGOs or public service departments to organize trainings and campaigns. Compared to conventional peer support groups explored by previous studies, DWC HK appears to be better informed by and aligned with formal support organizations. They are paving a way to enhance the accessibility of formal services and fill in the gaps when formal services or professionals lack cultural competence to support FDWs [30].

In the proactive posts, DWC HK admins frequently reminded new members about the types of professionals and organizations one can contact in certain circumstances. The results suggest that regular FDWs still need encouragement and guidance in identifying their problems and seeking or receiving proper help. For example, some FDWs may have been psychologically distressed when facing challenges like physical illness, unmanageable debts, losing important belongings (e.g., Example 8 in Table 1) or worrying about their family members in Philippines as a result of the pandemic or natural disasters (e.g., Example 7 in Table 1). However, they did not explicitly express the need for mental health support and instead focused on tangible support. Some FDWs only sought help when they could not endure harsh working conditions and asked for general advice on how they can survive, but did not realize that the employers might have violated their labor rights which should be protected by law. By contrast, the group admins, who often actively participate in activities organized by the Philippine Consulate and local NGOs, are more aware of the relationship between mental health and labor rights and have been acting like agents of change to influence their peers through those proactive posts.

### 4.3. Complete and Incomplete Support

While most of the needs explicitly expressed by individual FDWs were well attended to by the online group, including the group admins and other peer members, some needs might have exceeded the group’s capacity. For example, to look for a missing person would require police investigation, and to find a medical specialist and proper medical insurance would require in-depth knowledge about the local healthcare landscape. Moreover, due to their long working hours, admins and peer members are not always able to follow-up to check if the inquiries have eventually been resolved. Nonetheless, the results show that the posts containing unaddressed needs received more shares and reactions than those which were addressed. This suggests that when a certain issue cannot be addressed by the group, group members might have tried to ask for external help by sharing the posts or showing moral support by reactions.

As shown in Examples 9 and 11 on Table 1, although the page founder has carried out a tremendous job to provide support to her peers, the tension between her very limited spare time and the increasing demand for help might have put a psychological burden on her. In addition, as indicated by Example 12 in Table 1, the group experienced a split lately due to disagreements and a power struggle among the group admins. It is challenging even for formal organizations to manage their group dynamics, and similar power struggles have happened to some conventional peer support groups as well [16]. The group admins, especially the founder, may need external support such as consultations on self-care, leadership development, and group management. In addition, the group needs to recruit or train more peer volunteers to sustain the support, which can be strengthened through capacity-building programs and material support from formal support organizations, such as their hosting and home countries’ social welfare departments and NGOs.

### 4.4. Limitations

Because the online peer support groups for FDWs are relatively new and have rarely been engaged in research before, the present study is exploratory in nature. A few limitations of this study should be noted when interpreting the findings. Firstly, the study is only based on one online peer support group in HK. The extent to which the findings can be applied to other groups with the same nature is not clear. Nonetheless, this group had more than 120,000 followers when the data were collected. The size is greater than the number of respondents of any quantitative or qualitative studies conducted on FDWs in HK before. Therefore, the findings can complement previous studies utilizing conventional survey methods to research FDWs. Secondly, the analysis was based on four months of posts, which might have exhibited some temporary patterns that were bonded in that period of social context. We hope this study can encourage more studies to examine longer periods of data and more online peer support groups so that a bigger and clearer picture can be revealed. Thirdly, the social media metrics were used as indicators of the post impact in the analysis. Although this is a common practice in social media marketing research and can objectively measure FB users’ behaviors, it has limitations such as overlooking the impacts on silent FB users who only read posts but seldom leave comments or reactions. Similarly, the assessment of whether an expressed need was addressed was based on analyzing the public comments of a post. However, some cases might have been followed up by the group admins or members privately. Therefore, the result may have over-estimated the number of unaddressed needs. Future research can adopt population survey methods to measure the FB page’s followers’ perceived impacts, which can potentially compliment the present study’s methodological limitations. Fourthly, the final round of thematic content analysis was conducted by one coder, whose subjectivity may affect the results. Nonetheless, the coding scheme applied by the coder has been validated by two coders’ close discussions and a procedure of iterative coding comparison and refinement. The results were also triangulated by the other coder and key informants (i.e., founder and admins of DWC HK). 

### 4.5. Implications for Practice and Research

The present study’s findings warrant a proper recognition of the important role that online peer support groups can play in providing social support to FDWs. These groups should become working partners for formal support providers, such as social service and health service organizations, to better promote FDWs’ wellbeing. By monitoring these online groups and understanding their group dynamics and characteristics, formal support providers can better identify FDWs’ needs. Based on mutual understanding and agreement, collaborations can be carried out through online campaigns, education, and promotion, whereby those online groups can help outreach and connect FDWs with different needs to appropriate formal support. Formal support providers can learn from the online peer support groups by integrating emotional support with informational and instrumental support to enhance the service effectiveness and clients’ satisfaction level. Formal support providers should also help in sustaining this kind of online peer support groups by capacity building, quality checking, and sponsoring necessary resources, since these groups will in turn help formal support providers expand their accessibility and utility.

Since MDWs often live-in with their employers and engage in long working hours, previous research was challenged in the recruitment of participants and, therefore, view MDWs as a kind of hidden or hard-to-reach population. The present study sheds light on a new research arena to understand MDWs’ experiences, culture, and social networks. Compared to FDWs, MDWs from other countries often are even less resourceful [10,44]. Future research and practice should investigate whether a similar kind of online peer support groups also exist for MDWs from other countries and whether they share the same characteristics as the FDW group. Furthermore, future research should systematically examine the representativeness of those online groups’ participants to the overall MDW population, what commonalities and differences there are between those online groups and MDWs’ conventional social networks, and what new dimensions those online groups can add to our knowledge about MDWs’ culture, needs, group dynamics, and potential paths to wellbeing. 

## 5. Conclusions

Online peer support groups such as DWC HK have been actively providing all sorts of social support to their fellow domestic workers and achieving remarkable impacts. They are not only good at providing emotional support but also filling in service gaps in terms of informational and instrumental support. The group admins are acting as agents of change to spread knowledge and skills that they learned from formal support resources to a broader FDW community. However, they also face challenges such as limited capacity and unstable group dynamics. Formal support services and professionals should recognize and collaborate with them to support their long-term sustainability.

## Figures and Tables

**Table 1:** Posts attending to different social support needs in the Facebook peer-support group.

	Definition	Examples	Number of Posts	Median Number of Comments per Post (Range)	Median Number of Shares per Post (Range)	Median Number of Reactions per Post (Range)
Informational support (IM)	Posts sharing new information, news, perspectives, advice, references to other resources (i.e., support offered by other people or organization but not DWC HK), and appraisal support.	(1) “The OEC website no longer exists. BMOnline (is the new one). How to Register on the new website (Let’s Register) Step 1: Click this link https://onlineservices.poea.gov.ph/OnlineServices/POEAOnline.aspx? (accessed on 2 April 2022). Follow the pics for now please”* (Note: OEC refers to Overseas Employment Certificate). (2) “If you need counselling, please message Okayminds (Note: Okayminds is a local NGO). If you have friends who tell you they are experiencing stress, anxiety, or depression, refer them to help immediately”. *(Note: a link to and a photo of Okayminds’ Facebook page is attached).	324	73 (0~1000)	136 (0~1171)	472. 5(0~3127)
Emotional support (EM)	Posts providing encouragement, comfort, care, companionship, affinity, and/or acceptance to MDWs through the content, including text, pictures, or videos.	(3) “Let’s just laugh for now 🤣🤣🤣😂😂😂😂”.* (Note: A link to a funny YouTube video is attached). (4) “Let me share a beautiful photo I have taken. Comment below ⍗⍗⍗♡♡♡ How beautiful ♡♡♡ Credit to ### (Note: a name being de-identified by the researchers) I saw her post in the comment section of the attendance-taking”. * (Note: A photo of the sky taken by a member of DWC HK is attached).	143	205.5 (0~2100)	208 (0~575)	1133 (0~3475)
Instrumental support (IT)	Posts offering financial aid, material resources, and needed services directly from the DWC group, including offering hands-on help in getting necessary tasks carried out (e.g., matching loss with found), providing something of use (e.g., winter clothes donation and distribution), or performing emergency assistance (e.g., a hotline offered by DWC HK).	(5) “Found wallet ILLORE. ANIE BERDIN at T-shop. One member posted about it”. * (6) “For those who have messaged, please provide this location. Thank you so much for your new winter clothes. For those who do not have any, do come”. *(Note: In December 2021, some admins and members of DWC HK organized a winter clothes drive to distribute clothes to MDWs who do not have any).	49	4.5 (0~497)	18 (0~137)	108 (0~502)
IM, EM	__	(7) “Please help sender who will be going home this week…Thanks for the help. (From sender): Good morning admin, can I ask for help, I need to go home because of an emergency. What do I need to do and what papers do I need to go home quickly? My child passed away. Thanks for the reply. (From admin): Our condolences”. * (8) “Sender needs encouragement, what should be done? We have already talked, reported to the police, and viewed the CCTV of the building. Maybe you have other ideas. (Below from sender:) Good morning Mam/Sir I’m asking for help because last Monday Nov 8 my balikbayan box (Note: “Balikbayan boxes” (lit. repatriate box) are parcels that MDWs would spend a long time filling up until they can send it to their families at the end of the year) was picked up (by someone) in our building. I thought they were the Cargo company because I know they would pick it up on Monday. Then I paid for Mon. Nov 14. Only after 2 days Nov 16 they told me that they don’t have my box. I filled up the box. I will report to the police. Please help me please… I’m hoping I can find my box. For my family’s Christmas.I. (Below from admin:) For those who are waiting for their box to be picked up, put the box inside your house or keep an eye on the box yourself and keep the signed receipt when they pick it up. Lovely”. *	49	89 (0~740)	446 (0~806)	1613 (16~2965)
IM, IT	__	“Just a reminder to all the new followers of DWC, I am a Domestic Helper too. If I can’t answer the message on the page, there are still guides who can still help. DWC hotline 91613074 CALL—I can answer if my employer is not there even though they are kind, NO PHONE for me unless I finish my work. TEXT Messages—sometimes I don’t have load so if I don’t reply, please WhatsApp me. Whatsapp—Everyday I receive 80–120 messages, so when I know I can’t answer, I don’t check the messages because it’s hard to respond or find help. If you need help and if we don’t respond to you immediately, look for someone who can help you. Thank you so much. Lovely” * (9) For better services of DWC DWC Hotline 46267746/91613074 Mission whatsapp—95292326 HELP whatsapp—54934660 DSWD—28238537 POLO—55291880 MISSION HOTLINE—25228264 HELP HOTLINE—25234020 CONSULATE—91554023 OWWA—63459324 PATHFINDERS—51904886 “if you know someone needs help, I hope you know (these contact numbers) and give it to them”. * (10) “For those who are confused about visa, especially those new OFWs in Hong Kong, send a photo of your visa to our WhatsApp at 91613074 u can cover the details but don’t cover the DATES send all your messages in one go, so that we can respond in one go. I don’t like it when you say “HI” and “Hello” and “can I ask a question”… I appreciate if you ask all the questions in one go so it’s quicker. For us to give you accurate information, we need to see a picture of your visa and TAKE NOTE there are other cases of passports that have expired and the date given by the immigration is also cut off. I will work first then reply to WhatsApp at 1 pm. Thank you. Mother Lovely” * (11) I am Rodelia M. Pedro, Founder of Domestic Workers Corner Hong Kong inform you that, starting (from) yesterday 9 November 2021 at 7:35 pm, the DWC HELP GROUP and DWC Lovely Corner Page are no longer part of Domestic Workers Corner HONG KONG. DWC includes only Domestic Workers Corner HONG KONG—Official Page, Lovely Corner, Foreign Domestic Workers corner, DWC GROUP, 💕IT’S ALL ABOUT FOOD💕, DWC LEARNING, and SOCIAL GROUP, DWC Yes To HOPE, DWC Saturday Group. Another new part of the Journey as I continue to serve the community 🙂 “thank you very much” * Rodelia/Lovely DWC hotline 91613074	25	9.5 (0~552)	27.5 (0~514)	268.5 (32~3842)
IT, EM	__	(12) “COVID changes everything. The situation of the Foreign Migrant Workers getting worse at their workplace and the demands of the employers Terminated cases increased, many visa denied and other issues Thanks to all NGOs as we collaborated with them for helping us in time of need and the Migrants Guide 2021 webinar will nearly end... Keep an eye out on Nov 29, 2021 at 12:30 pm Thank you so much Enrich HK” * (Note: Enrich HK is a local NGO. A link to this NGO’s FB page is included in the post).	7	28 (0~33)	4 (0~10)	316 (34~385)
IM, IT, EM	__	(13) “If we want to argue with our employer, let’s not give our notice immediately Let’s think first Do we have money to pay for a boarding house? Have we bought food? Do we have any debt to be paid? Can we support ourselves with our savings? Will our families be okay if we don’t send them money? Let’s think about how difficult it is to terminate our contracts now. Let’s also be firm so that we don’t get swayed by our emotions. I know it’s difficult but after arguing with your boss, take a break. Inhale exhale. Rest for a moment to calm yourself. When someone asks for advice, I say ‘I don’t want you to be terminated because it is difficult these days to have termination status.’ And then I give advice on what to do and report to NGOs because our work includes the employer’s attitude even if it is difficult to understand, it must be understood. No matter how difficult it is to live with, it must be lived with. I’m not a professional adviser but if there is a problem with the employer whatsapp me at 91613074 I will try to help no matter what. Plus I have a contact who can help, if you need someone professional Let’s be patient with our employers behaviour first. If we survived yesterday, we can survive today Pray always... God will give us patience and wisdom. God bless you Lovely” *	2	78.5 (1~156)	43 (8~78)	1368 (290~2446)

* Content in quotation marks was originally in Tagalog and translated by the researchers into English. All content in brackets was added by the researchers.

**Table 2:** Specific issues or circumstances that received support from the peer-support group.

	Total Number of Posts	Number of Posts Proactively Posted by the Admins	Number of Posts Posted per a Member’s Request
Daily life issues (e.g., exchange rates, directions to somewhere, news, or any other unspecified matters relevant to domestic workers)	111	89	22
Greetings, prayers, sharing of funny or heartwarming things	90	90	0
Update about regular activities among the group members (e.g., Christmas gathering, Sunday gathering, winter clothes donation, etc.)	77	74	3
Physical health (e.g., health check, HIV/AIDS test, COVID-19 test or vaccination, etc.)	72	59	13
Legal documents (e.g., HK ID application, work visa renew, contract termination, etc.)	50	46	4
Missing belongings or person	32	0	32
Skills sharing and training (e.g., cooking skills, skills of taking care of person with disabilities, etc.)	28	26	2
Employer-related (e.g., help employer to find a new domestic worker, how to better communicate with employers, etc.)	22	10	12
Reminder about scams or crimes	20	18	2
Political participation (e.g., overseas voter registration, Philippine elections, etc.)	19	19	0
Financial literacy	17	16	1
Mental health	10	9	1
Labor rights (e.g., statutory holidays, equal opportunity of pregnancy, advocacy for social inclusion, etc.)	8	8	0
Community member’s death	4	4	0
Total	599	509	90

**Table 3:** Comparison between expressed needs that were addressed or not by the online peer-support group.

	Number of Posts	Median Number of Comments (Range)	Median Number of Shares(Range)	Median Number of Reactions (Range)
**Addressed**	45	285.5 (4~908)	5.5 (0~202)	313 (15~1161)
**Not addressed**	13	129 (0~740)	11 (0~211)	524.5 (0~1867)

## Data Availability

The data presented in this study are available on request from the corresponding author. The data are not publicly available due to copyright concerns.
